# Demonstrating persistence of foot-and-mouth disease virus in African buffalo (*Syncerus caffer*) using BaseScope™ in situ hybridisation

**DOI:** 10.1007/s11259-025-10898-3

**Published:** 2025-09-23

**Authors:** Alischa Henning, Lieza Odendaal, Angelika Loots, Melvyn Quan

**Affiliations:** 1 Department of Paraclinical Sciences, Faculty of Veterinary Science, University of Pretoria, Pretoria, South Africa; 2 Department of Veterinary Tropical Diseases, Faculty of Veterinary Science, University of Pretoria, Pretoria, South Africa

**Keywords:** *Aphthovirus vesiculae*, BaseScope™, Buffalo, Carrier, Foot-and-mouth disease, In situ hybridisation, RNA, Viral persistence

## Abstract

BaseScope™ in situ hybridisation (Advanced Cell Diagnostics, USA) was used to detect foot-and-mouth disease virus (FMDV), species *Aphthovirus vesiculae*, in formalin-fixed paraffin-embedded tissues from African buffalo (*Syncerus caffer*, *n* = 15) culled from the Kruger National Park - where the South African territories (SAT)-1, -2 and − 3 serotypes are endemic - as part of their population management program. Foot-and-mouth disease viral RNA was consistently detected in the palatine tonsils and lungs, demonstrating these as primary sites of viral persistence. Detection in the retropharyngeal lymph nodes and interdigital skin was less frequent, while oropharyngeal tissue showed rare positivity. Other sampled tissues - including the tip of the ear, eyelid, tongue, lip, and coronary band - proved suboptimal for identifying FMDV-positive buffalo or carriers. These findings highlight the value of BaseScope™ for detecting low viral loads of FMDV in persistently infected African buffalo, with a notable predilection for the palatine tonsils and lungs.

## Introduction

Foot-and-mouth disease virus (FMDV) is a single-stranded, positive-sense RNA virus of the genus *Aphthovirus* (family *Picornaviridae*), that causes foot-and-mouth disease (FMD), a highly contagious disease affecting both domestic and wild cloven-hoofed animals. Transmission occurs between infected and susceptible animals - by inhaling infected aerosols, exposure to contaminated fomites, and pigs consuming contaminated animal products (Gloster et al. 2006; Pacheco et al. 2010; Brown et al. 2022). Whilst clinical disease in cattle include pyrexia, vesicular lesions, and lameness, African buffalo (*Syncerus caffer*) typically have subclinical infections and serve as long-term carriers, particularly of the Southern African Territories (SAT)−1, −2, and − 3 serotypes prevalent in southern Africa (Bronsvoort et al. 2008; Di Nardo et al. 2015; Lazarus et al. 2018; Perez-Martin et al. 2022). Carrier animals maintain the virus in the population, complicating control and eradication efforts (Salt 1993; Vosloo et al. 2002). Viral detection in these animals is challenging due to low and inconsistent viral loads in tissues.

According to the World Organisation for Animal Health (WOAH), FMD must be diagnosed by detecting/demonstrating FMDV, viral antigen or viral nucleic acid. Standard diagnostic techniques such as RT-PCR and virus isolation, the FMD diagnostic tests of choice, require high-containment biosafety level 3 (BSL-3) facilities. Formalin-fixed tissues, however, allow for downstream molecular diagnostics in lower biosafety settings. In situ hybridisation (ISH) techniques, including the novel BaseScope™ RNA-ISH, enable spatial localisation of viral RNA in tissue sections and offer enhanced sensitivity and specificity compared to traditional ISH.

Given the prevalence of the SAT serotypes in South Africa and the role of African buffalo as key maintenance hosts and important carrier animals, understanding the tissue distribution and persistence of FMDV in these animals is crucial for effective disease control. This is particularly true in endemic areas such as the Kruger National Park (KNP) where serological studies have shown that up to 98% of buffalo exhibit antibody responses to all three SAT serotypes (Thomson et al. 1992; Thomson 1997). To investigate the presence of FMDV in buffalo tissues BaseScope™ was selected for its exceptional sensitivity and specificity which stem from patented signal amplification and background suppression systems that reduce non-specific staining and enables detection of short RNA targets (Neau et al. 2019). The specificity of this assay is achieved through the requirement for two ‘double-Z’ target probes to contiguously bind to their respective complementary RNA sequences. This allows for the subsequent binding of the preamplifier, initiating a signal amplification cascade. This ‘double-Z’ binding ensures low to no background signal. This makes it especially suited for detecting conserved serotype-specific regions of FMDV which are often short. Unlike traditional ISH techniques, BaseScope™ does not require an RNA-free environment and permits visualisation of viral RNA in formalin-fixed paraffin-embedded tissues sections. A custom probe can be based on the Callaghan reference sequence targeting a highly conserved region of the viral genome that can detect all three SAT serotypes. These attributes are valuable in detecting low viral loads expected in carrier animals.

Although RNAscope™ and, to a lesser degree, BaseScope™ technology have been applied to various genes, tissues, and species, it has rarely been applied to wildlife and has not been used on African buffalo tissue. In addition, there is a single, recent report where RNAscope™ was applied to detect FMD in carrier cattle (Litz et al. 2024). Therefore, the aim of this study was to apply BaseScope™ RNA-ISH to formalin-fixed paraffin-embedded tissues (FFPE) from African buffalo known to be FMDV carriers. A further aim was to characterise tissue tropism and improve our understanding of virus persistence in wildlife reservoirs.

## Materials and methods

### Analytical controls

The conditions for the ISH assay were first optimised using FMDV-positive and negative FFPE control blocks prepared with the assistance of staff from the Transboundary Animal Disease Laboratory of the Agricultural Research Council-Onderstepoort Veterinary Research (ARC-OVR).

A monolayer of baby hamster kidney (BHK)−21 cells (ATCC, Manassas, VA, USA) were grown in Glasgow’s minimum essential medium (MEM) (Gibco™, ThermoFisher Scientific, Waltham, MA, USA) containing 5% foetal bovine serum and 1 × penicillin-streptomycin-glutamine antibiotic supplements in 75 cm^2^ cell culture flasks (Corning™, Corning, New York, USA). Three positive control flasks were inoculated at the multiplicity of infection (MOI) ratios of 0.1, 1.0, and 2.5 with SAT 3 FMDV (KNP 10/90/3) (ARC-OVR), whereas the negative control flask remained uninoculated. Cells were incubated at 37 °C and 5% CO_2_ for approximately 13 h. More prolonged incubation resulted in advanced cytopathic effect (CPE) and detachment of a large proportion of cells. At 13 h, CPE corresponding with MOI was observed and recorded for all flasks. Cells were then trypsinised with trypsin-EDTA and harvested in RPMI medium containing 10% foetal bovine serum (ThermoScientific™, ThermoFisher Scientific, Waltham, MA, USA). Harvested cells were suspended in 4% paraformaldehyde and fixed by 15 min incubation at room temperature (RT) before they were rewashed twice with phosphate-buffered saline (PBS) and suspended in prewarmed HistoGel™ (ThermoScientific™, Richard-Allan Scientific). The samples were kept at 4 °C overnight to allow the HistoGel to solidify. The fitness for use of the prepared FFPE cells as positive and negative controls was confirmed by triplicate RT-qPCR of cell block sections (Heid et al. 1996). The harvested cells suspended in the HistoGel™ were then transferred to the histopathology laboratory, Section of Pathology, Department of Paraclinical Science, Faculty of Veterinary Science (FVS), University of Pretoria (UP), South Africa and embedded into paraffin blocks.

Positive and negative control blocks were included in each ISH run, incorporating tissues from the positive control bovine, test buffalo, and negative control buffalo. Given the rarity of clinical FMDV infection in buffalo and the associated paucity of infected cells, these controls helped account for low viral titres and validated both positive and negative ISH results.

### Diagnostic samples

Positive diagnostic control tissues from adult, female, crossbreed cattle (*n* = 6) experimentally inoculated with FMDV (SAT2 KNP/1/10 and ZIM7/83/2) were sourced. They were slaughtered approximately ten days post infection. The bovine (*n* = 1) with the lowest cycle threshold (C_t_) of 29 was used as the positive control since this suggested a high target concentration.

Tissues used in the current study were obtained from African buffalo (*n* = 30) culled/slaughtered in the KNP. From this, 15 represented the test samples and 15 the negative control samples. The test buffalo (*n* = 15) were culled as part of the park’s population control/harvest management program. The FMD statuses of the buffalo were determined by RT-PCR performed on serum samples at the ARC-OVR as previously described (Callahan et al. 2002; Reid et al. 2003). Serum samples for RT-PCR were collected at the time of slaughter and subsequent tissue sampling, while PCR analysis was performed at the ARC-OVR. This assay can detect viral RNA in less than two hours and is a reliable, rapid and sensitive diagnostic test for the detection of FMDV (Callahan et al. 2002). Based on WOAH guidelines, buffalo with a C_t_ lower than 40 were categorised as positive. Serotyping was not conducted, as African buffalo in the KNP are frequently co-infected with multiple serotypes (Bronsvoort et al. 2008; Maree et al. 2014, 2016; Di Nardo et al. 2015; Lazarus et al. 2018). Historical data from the ARC-OVR, the reference laboratory for FMD, confirm that SAT-1, SAT-2, and SAT-3 are endemic in this population, with concurrent infections commonly observed. The negative control tissues (*n* = 15) repeatedly tested negative for FMDV by RT-PCR. These buffaloes were PCR-negative for FMDV at 30 to 60-day intervals for three rounds, with the final round coinciding with the slaughter date. All animals included were female and were subadults, aged between two and four years of age.

The following tissue samples were collected from all the animals (positive control bovine, negative control buffalo and test buffalo) included in this study: tip of the ear (left or right), eyelid (left or right), lip (top or bottom), tongue, oropharyngeal tissue, retropharyngeal lymph nodes (medial and lateral), palatine tonsil, lung (left and right), coronary band (all four limbs) and interdigital skin (all four limbs). Post collection, the samples were placed in 10% buffered formalin and transported to the histopathology laboratory, FVS, UP, South Africa. Formalin-fixed tissues were processed to FFPE blocks within 7–14 days post-collection. The tissue samples were processed, embedded and sectioned according to the Department of Agriculture-accredited standard operating procedures. To avoid repeated trimming of blocks and minimise the loss of tissues for follow-up studies, all the blocks were cut into four µm thick sections until the blocks were too thin to cut any more sections. Cut sections were floated in warm water, picked up onto microscope slides, dried, and stored for seven days to three months. One set of slides per animal was stained according to the Department of Agriculture-accredited standard operating procedures for haematoxylin and eosin (HE)-stained sections for the histopathology evaluation. The HE-stained sections were scanned with a Motic Easyscan Pro 6 Digital Slide Scanner. The digital images were evaluated using the Motic DSAssistant digital software.

#### In situ hybridisation assay

##### Probe design

A custom BaseScope™ probe designed by ACD was used (a 1ZZ probe named BA-V-FMDV-SAT2-pp-1zz-st targeting 3969–4014 of KJ144904.1). The probe targeted SAT2/KNP/51/93 P2/P3 polyprotein gene, partial cds (Callahan 3D P TCCTTTGCACGCCGTGGGAC (4015–4034)). Custom probe specificity was ensured through a combination of bioinformatics, commercial design algorithms and testing with positive and negative probes and controls under optimised conditions. In Silico analysis confirmed that this probe could detect approximately 197 viral sequences across SAT-1, SAT-2 and SAT-3 serotypes thereby enhancing utility for broad detection in endemic buffalo populations.

##### In situ hybdridisation

A novel ISH assay (BaseScope™ Technology, Advanced Cell Diagnostics, Hayward, California, USA) was used. The Advanced Cell Diagnostics HybEZ II Hybridization System (supplied by Whitehead Scientific) was used with the BaseScope™ kit and custom probe per the manufacturer’s instructions. This system included an oven, humidity control tray, slide holder, wash tray and humidifying paper. The pre-treatment, target retrieval, hybridisation and detection was performed as previously described and optimised (Henning et al. 2025). The probe was applied to the tissue samples, hybridised overnight, and the signal was observed using light microscopy.

### Interpretation

Histopathology was descriptive and focused on the presence and details of any FMD-related lesions. The ISH results were dichotomised as positive or negative for the presence of FMDV. A light microscope (Olympus BH-2) was used to determine the presence of labelling compared to the PCR results per case. Positive labelling consisted of the presence of bright pink punctate dots. All the cases were evaluated by an experienced observer (Veterinary Anatomic Pathologist) over a limited number of days to decrease observer variability and bias. Thereafter, the ISH for FMDV were scored on an ordinal scale as negative (-/0), equivocal (±/1) (a slight increase over the background), isolated (+/2), scattered positive (++/3) or widespread (+++/4). The arithmetic mean was calculated by treating the ordinal values as numerical scores. Ten high power fields per tissue section were evaluated to obtain the specific score. The intensity of the immunoreactivity was not included within the scope of the work, since the ISH was performed manually. Pearson/Spearman correlation was used to draw inference between the obtained values.

## Results

**Fig. 1:**
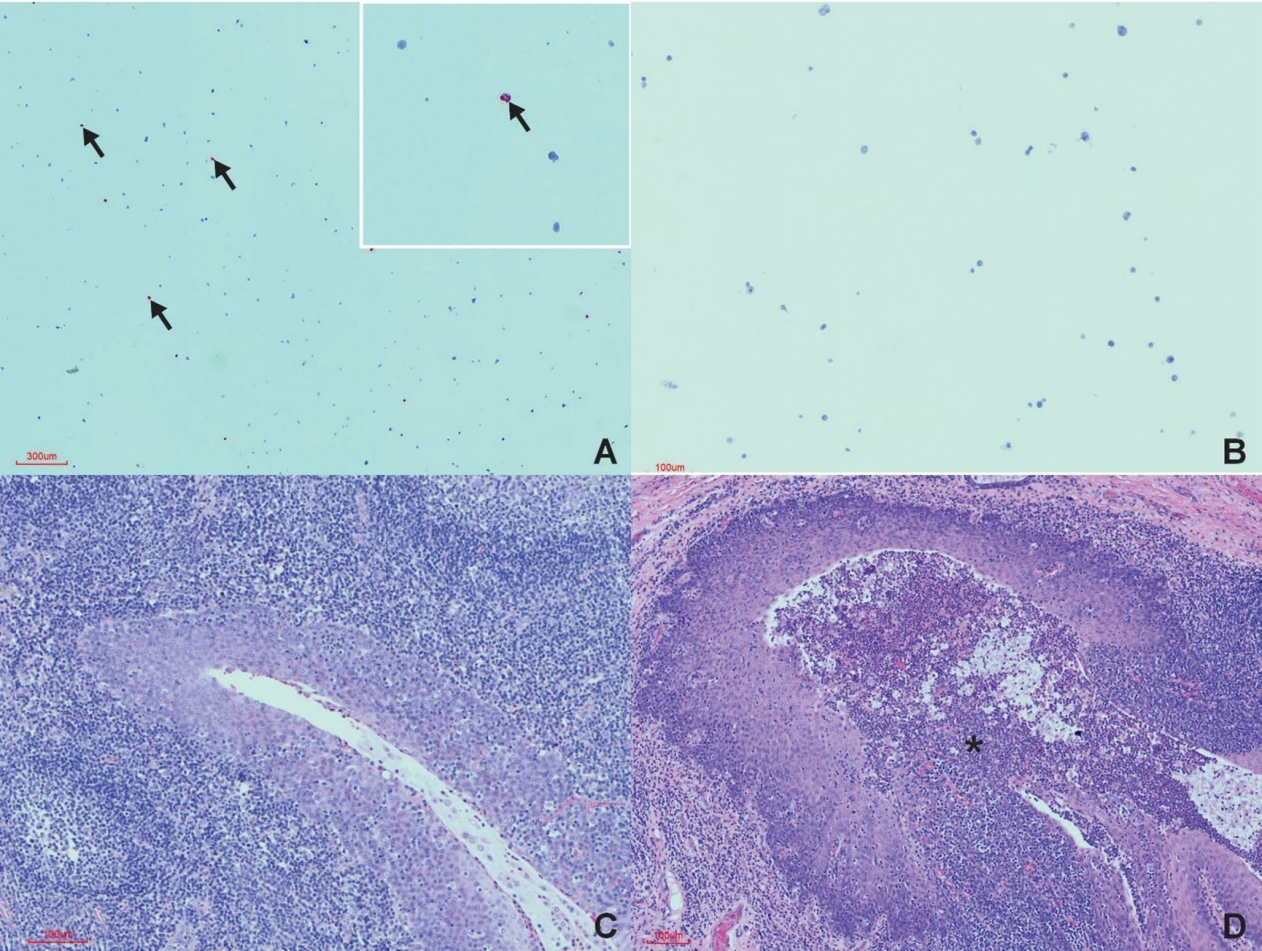
BaseScope™ ISH results and histopathology in FMDV-positive and negative cell culture controls (**A **and** B**), FMDV-positive carrier buffalo (**C**) and FMDV-positive bovine (**D**). (**A**) Fine red granules (*line arrows*) in a positive cell culture section (MOI = 2.5) (**B**) No staining in a negative cell culture section (**C**) Palatine tonsil, HE-stained section of ISH-positive, PCR-positive carrier buffalo with no appreciable microscopic lesions associated with the tonsillar crypt epithelium and neighbouring lymphoid follicles) (**D**) Palatine tonsil, HE-stained section of PCR-positive bovine with a crypt abscess (*star*) and expanded crypt epithelium as compared to (**C**)

**Fig. 2:**
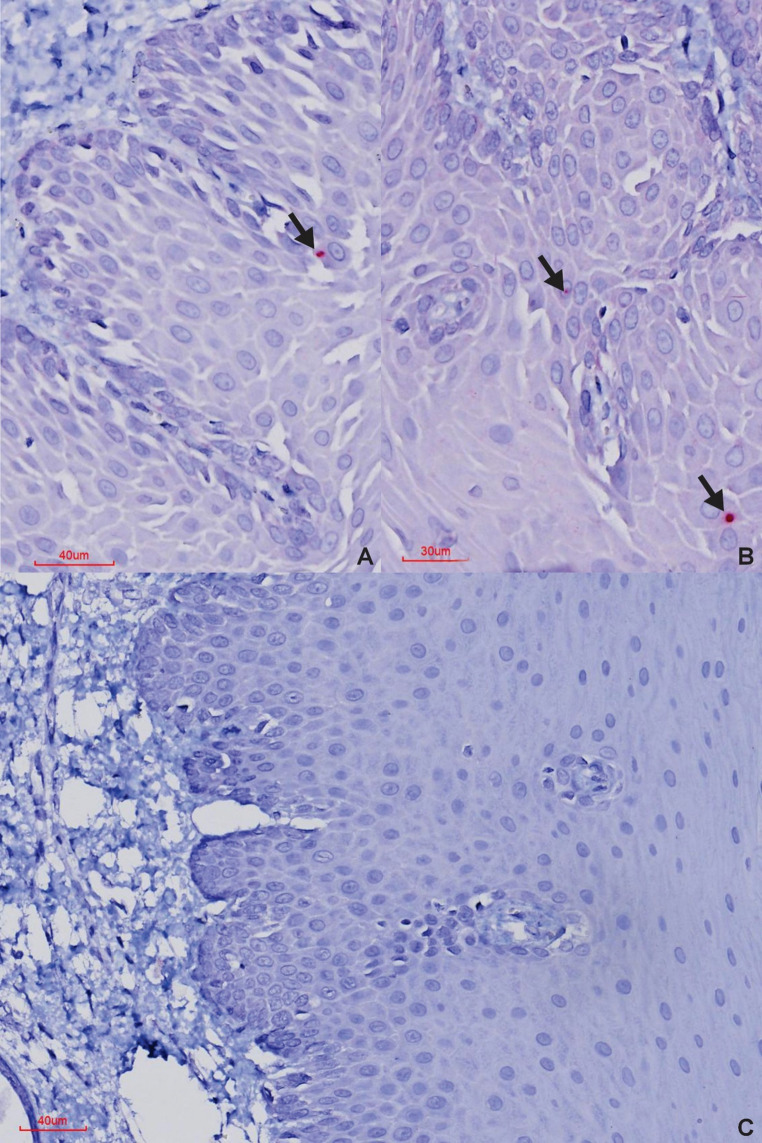
BaseScope™ ISH results in FMDV-positive carrier buffalo (**A **and** B**) and FMDV-negative buffalo (**C**). (**A**) Palatine tonsil and (**B**) interdigital skin, ISH-positive sections of PCR-positive buffalo with positive labelling in the overlying epithelium (*line arrows*) (**C**) Palatine tonsil, ISH-negative sections of PCR-negative buffalo with no positive labelling visible

**Fig. 3:**
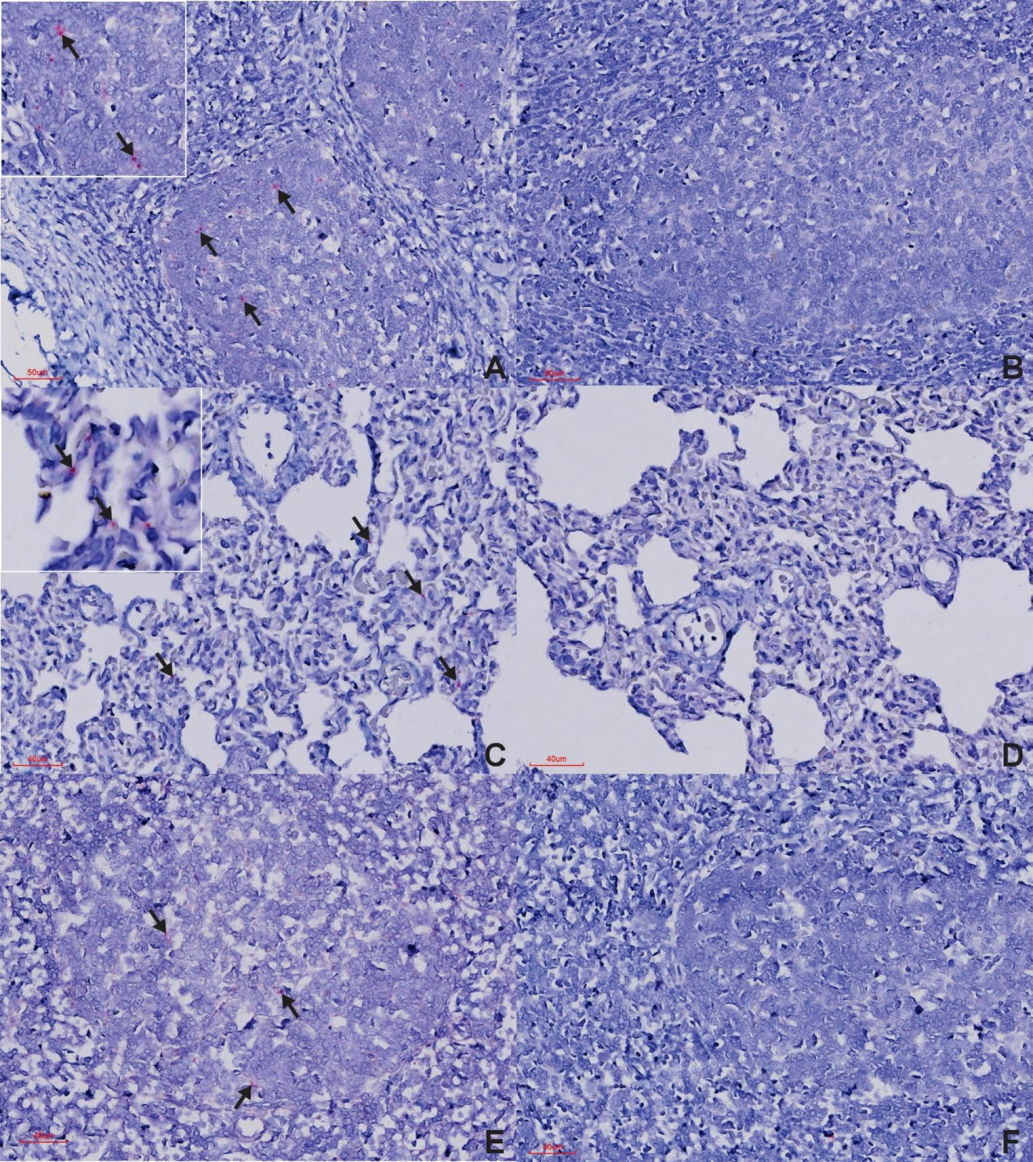
BaseScope™ ISH results in FMDV-positive carrier buffalo (**A**,** C **and** E**) and FMDV-negative buffalo (**B**,** D **and** F**). (**A**) Palatine tonsil, ISH-positive section of PCR-positive buffalo with positive labelling of germinal centres with inset at higher magnification (*line arrow*) (**B**) Palatine tonsil, ISH-negative section of PCR-negative buffalo with no positive labelling visible (**C**) Lung, ISH-positive section of PCR-positive buffalo with positive labelling located to the alveolar walls with inset at higher magnification (*line arrows*) (**D**) Lung, ISH-negative section of PCR-negative buffalo with no positive labelling visible (**E**) Retropharyngeal lymph node, ISH-positive sections of PCR-positive buffalo with positive labelling within the germinal centres (*line arrows*) albeit to a lesser extent than noted in palatine tonsils (**F**) Retropharyngeal lymph nodes, ISH-negative section of PCR-negative buffalo with no positive labelling visible

The FMDV-negative control block and buffalo tissues consistently showed no ISH-positive labelling across all batches (Figs. 1, 2 and 3). No viral RNA was detected in any of the evaluated tissues, including the tip of the ear, eyelid, lip, tongue, oropharyngeal tissue, retropharyngeal lymph nodes, palatine tonsils, lungs, coronary band and interdigital skin. Histologically, there were no appreciable lesions in any of the negative control buffalo tissues (Fig. 1).

The FMDV-positive control block consistently exhibited ISH-positive labelling across all batches, validating assay performance (Fig. 1). Additionally, the blocks with MOI rations of 0.1, 1.0 and 2.5 were all equally effective as controls. Positive ISH labelling was restricted to oropharyngeal and palatine tonsillar tissues in FMDV-positive bovine tissue. The labelling was localised primarily to lymphoid germinal centres and, to a lesser extent, the overlying epithelium. Histopathological findings in the palatine tonsils included crypt abscesses composed of necrotic debris, fibrin, and a mixed inflammatory infiltrate (predominantly lymphocytes with occasional neutrophils), along with lymphocyte migration across the epithelium and mild to moderate lymphoid depletion (Fig. 1). Other tissues showed non-specific changes, including pulmonary congestion, oedema, and haemorrhage.

In the PCR-positive test buffalo, ISH consistently detected viral RNA in the palatine tonsils and lungs, with less frequent detection in the retropharyngeal lymph nodes, interdigital skin, and rarely in the oropharyngeal tissue (Tables 1 and 2; Figs. 2 and 3). Positive labelling in the palatine tonsils and retropharyngeal lymph nodes was predominantly confined to the lymphoid germinal centres, with some signal in the overlying epithelium. In the lungs, labelling was evident within the alveolar walls, while in the interdigital skin, signal was observed in the superficial epithelium (Figs. 2 and 3). No ISH signal was detected in the tip of the ear, eyelid, lip, tongue, or coronary band in any test buffalo. Additionally, test buffalo numbers two and six did not show ISH-positive labelling in any of the tissue examined. Microscopically, none of the test buffaloes showed any appreciable lesions.

The images presented in the figures are representative and do not reflect all cases examined.

**Table 1:** Outcome of ISH per location in test buffalo group. The presence of FMDV was scored on an ordinal scale as negative (-/0), equivocal (±/1) (a slight increase over the background), isolated (+/2), scattered positive (++/3) or widespread (+++/4). There was no ISH-positive labelling in tip of the ear, eyelid, lip, tongue or coronary band of any test buffalo

Test buffalo No	PCR result		ISH result						
	Result	Ct value	Combined result for all tissues	Palatine tonsils	Lungs	Retro-pharyngeal lymph nodes	Inter-digital skin	Oro-pharyngeal tissue	Tissue mean^*^
1	Positive	33.39	Positive	+ (2)	+ (2)	± (1)	± (1)	− (0)	1.6
2	Negative	> 40	Negative	− (0)	− (0)	− (0)	− (0)	− (0)	-
3	Positive	36.62	Positive	+ (2)	+ (2)	+ (1)	+ (1)	− (0)	1.6
4	Inconclusive	39.98	Positive	+ (2)	+ (2)	+ (1)	± (1)	− (0)	1.4
5	Positive	33.69	Positive	± (1)	+ (2)	+ (1)	± (1)	− (0)	1.2
6	Negative	> 40	Negative	− (0)	− (0)	− (0)	− (0)	− (0)	-
7	Positive	32.14	Positive	± (1)	+ (2)	+ (1)	± (1)	± (1)	1.4
8	Positive	39.01	Positive	+ (2)	± (1)	± (1)	± (1)	− (0)	1
9	Positive	33.16	Positive	+ (2)	+ (2)	+ (1)	± (1)	− (0)	1.4
10	Positive	36.84	Positive	+ (2)	+ (2)	± (1)	± (1)	− (0)	1.2
11	Positive	37.69	Positive	++ (3)	+ (2)	+ (1)	± (1)	− (0)	1.6
12	Positive	36.84	Positive	+ (2)	± (1)	+ (1)	± (1)	± (1)	1.4
13	Positive	33.06	Positive	+ (2)	± (1)	± (1)	+ (1)	± (1)	1.4
14	Positive	35.77	Positive	+ (2)	+ (2)	± (1)	± (1)	− (0)	1.2
15	Positive	35.56	Positive	+ (2)	± (1)	± (1)	± (1)	− (0)	1
Control buffalo									
1–15	Negative	> 40	Negative	− (0)	− (0)	− (0)	− (0)	− (0)	-

^*The arithmetic mean was calculated by treating the ordinal values as numerical scores^

**Table 2:** Summary of quantitative FMDV ISH-positive labelling of various organs including palatine tonsil, lungs, retropharyngeal lymph nodes, interdigital skin and oropharyngeal tissue of test buffalo group. There was no ISH-positive labelling in tip of the ear, eyelid, lip, tongue or coronary band of any test buffalo

Tissue	No of animals with FMDV-positive ISH labelling/No of animals with PCR-positive result	ISH score range	Tissue mean
Palatine tonsil	13/12	0–3	1.67
Lungs	13/12	0–2	1.47
Retropharyngeal lymph nodes	13/12	0–2	1.33
Interdigital skin	13/12	0–2	1
Oropharyngeal tissue	3/12	0–1	0.2

**Fig. 4:**
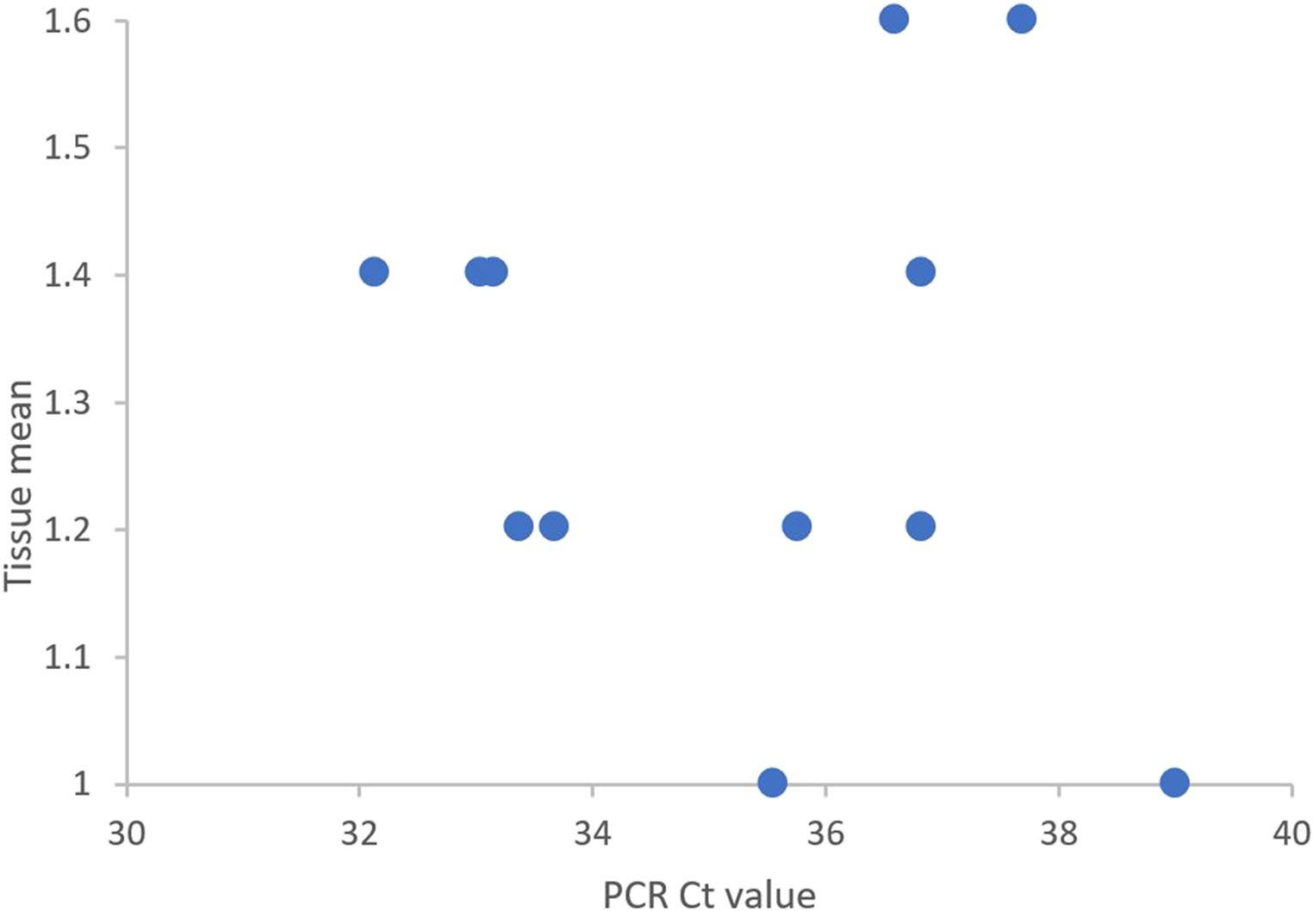
Correlation between PCR values and tissue mean

No statistically significant correlation (Fig. 4) was observed between the PCR result and the Tissue mean in Table 1.

## Discussion

The persistence and tropism of FMDV in African buffalo (*Syncerus caffer*), a recognised carrier species, was investigated using a novel BaseScope™ ISH assay. Consistent ISH-positive labelling was observed in the palatine tonsils and lungs, followed by less frequent detection in the retropharyngeal lymph nodes and interdigital skin. Oropharyngeal tissue revealed rare positivity, and no viral RNA was detected in other sampled tissues, including the tongue, lip, eyelid, ear tip, and coronary band.

Given the low viral loads in clinically recovered or asymptomatic animals, positive and negative control blocks were included in each ISH batch to validate both true-positive and true-negative results. Despite confirmed FMDV presence, neither the test buffalo nor the positive control bovine exhibited appreciable microscopic lesions. This is consistent with previous reports suggesting that microscopic pathology is absent in carrier buffalo and more so beyond the acute viraemic phase - typically within 48–72 h post-infection (Murphy et al. 1999; Alexandersen et al. 2002; Arzt et al. 2010; Maree et al. 2016; Cortey et al. 2019; Perez-Martin et al. 2022; Sahoo et al. 2022). The absence of lesions in carrier animals is hypothesised to result from non-lytic viral replication, which may reduce the need for a robust immunologic response (Salt 1993).

African buffalo can be carriers for extended periods with an average of five years and up to 20 years in isolated herds (Arzt et al. 2011). Of the 15 buffalo examined, 12 were PCR-positive and 13 were ISH-positive, aligning with serological studies showing widespread FMDV exposure among KNP buffalo (Thomson et al. 1992; Thomson 1997; Bronsvoort et al. 2008; Maree et al. 2014, 2016; Di Nardo et al. 2015; Lazarus et al. 2018). Despite extensive research, the role of carrier animals in FMDV transmission remains unclear. Although virus titres in carriers tend to decline to low or undetectable levels, consistent viral detection in the lung tissue may support a possible role in airborne transmission and long-term maintenance of the virus in endemic ecosystems (Donaldson and Kitching 1989; Juleff et al. 2008).

BaseScope™ identified viral RNA predominantly in germinal centres of the palatine tonsils and retropharyngeal lymph nodes, with occasional epithelial labelling. In the lungs, viral RNA was localised to the alveolar walls and in the epithelium of the interdigital skin. The precise cellular identity of the infected cells in the lung could not be determined; however, both alveolar macrophages and type II pneumocytes remain plausible candidates, with implications for systemic versus epithelial viral persistence.

Despite existing literature identifying the oropharyngeal region as a primary site of FMDV persistence in carrier animals and especially cattle, ISH in this study revealed no positive labelling in the oropharyngeal tissue of most of the test buffalo. Several plausible explanations may account for this discrepancy. First, the viral load in this tissue may have declined below the detection threshold due to the temporal dynamics of carrier-state persistence, especially if infection occurred long before sampling (Juleff et al. 2008; Arzt et al. 2011). Second, sampling may have missed focal sites of viral persistence, as FMDV distribution within the oropharynx is known to be patchy (Zhang and Kitching 2001; Alexandersen et al. 2002). Furthermore, local immune-mediated clearance in the oropharynx may result in site-specific viral elimination while allowing persistence in other tissues such as the lungs or tonsils (Thomson et al. 1992; Cortey et al. 2019). However, this requires further study to confirm. Lastly, current assumptions about oropharyngeal persistence are largely based on cattle studies, and tissue tropism in African buffalo may differ substantially. Our findings, consistent with Maree et al. (2016), suggest preferential viral persistence in lymphoid tissues over associated epithelium in carrier buffalo.

BaseScope™ ISH offers distinct advantages in detecting FMDV in carrier African buffalo. While RT-PCR remains a sensitive tool for viral RNA detection, it provides no spatial information. In contrast, BaseScope™ allows direct visualisation of viral RNA within specific tissue structures and cell types, enabling precise localisation of viral persistence. In this study, BaseScope™ identified consistent viral presence in the palatine tonsils and lungs - findings that were not reliably predicted by RT-PCR alone. This highlights the importance of using these diagnostic modalities not as direct comparisons, but as complementary tools; RT-PCR excels in detecting low-level viral RNA whereas BaseScope™ reveals anatomical and cellular context. Furthermore, the ability of BaseScope™ to operate effectively on FFPE tissues and to detect short or degraded RNA sequences make it especially valuable for identifying virus in chronically or subclinical infected animals. These findings underscore the enhanced diagnostic and pathobiological insights RNA-ISH, can provide, particularly when studying elusive carrier states in wildlife reservoirs.

The custom probe, designed using the Callaghan reference strain, was validated to ensure broad applicability across SAT serotypes. The target region showed high conservation across roughly 197 serotypes spanning SAT-1, SAT-2 and SAT-3 providing confidence in its cross-detection potential. However, minor sequence divergence could potentially reduce hybridisation efficiency and lead to false negatives especially in samples with marginal RNA levels. This is particularly relevant in carrier buffalo where viral loads are expected to be low. Although BaseScope™ technology offers increased sensitivity, its ability to detect RNA still depends on adequate transcript abundance and sequence complementarity. Thus, the absence of signal in some tissues may not definitely indicate absence of virus but could reflect insufficient target RNA or mismatches between target and probe. In addition, tissue sampling depth may further influence detection outcomes especially if viral persistence is localised to specific microenvironments.

To our knowledge, this is the first study to comprehensively evaluate a broad panel of tissue types - including skin, mucosal, lymphoid, and pulmonary tissues - using RNA ISH for FMDV detection in African buffalo. Results suggest that, apart from palatine tonsils, the lungs are a reliable site for identifying carrier animals. Conversely, tissues such as the eyelid, ear tip, and lip yielded no diagnostic value. These findings refine the understanding of FMDV tissue tropism and viral persistence in carrier hosts and support the inclusion of pulmonary tissue in future diagnostic strategies. To build on these findings, future studies should incorporate RNAscope™ to enable broader serotype detection, longitudinal sampling to monitor the temporal dynamics of viral persistence, and multi-probe approaches to enhance diagnostic sensitivity in low-copy, carrier-state infections.

## Data Availability

The datasets generated and analysed during the current study are available from the corresponding author on reasonable request.
